# Prospective performance evaluation of selected common virtual screening tools. Case study: Cyclooxygenase (COX) 1 and 2

**DOI:** 10.1016/j.ejmech.2015.04.017

**Published:** 2015-05-26

**Authors:** Teresa Kaserer, Veronika Temml, Zsofia Kutil, Tomas Vanek, Premysl Landa, Daniela Schuster

**Affiliations:** a Institute of Pharmacy/Pharmaceutical Chemistry and Center for Molecular Biosciences Innsbruck, University of Innsbruck, Innrain 80-82, 6020 Innsbruck, Austria; b Laboratory of Plant Biotechnologies, Institute of Experimental Botany AS CR. v.v.i., Rozvojova 263, 165 02 Prague 6 – Lysolaje, Czech Republic; c Department of Crop Sciences and Agroforestry, Faculty of Tropical AgriSciences, Czech University of Life Sciences Prague, Kamycka 129, 165 21 Prague 6 – Suchdol, Czech Republic

**Keywords:** Method comparison, Docking, Pharmacophore modeling, Shape-based modeling, 2D similarity-based search, Cyclooxygenase, A, anion, AA, arachidonic acid, Acc, accuracy, ACE, angiotensin-converting enzyme, Ar, aromatic feature, C, cation, COX, cyclooxygenase, DES, diethylstilbestrol, DUD, Directory of Useful Decoys, ECFP4, Extended-connectivity fingerprints 4, EE, early enrichment, FCFP6, Functional-class fingerprints 6, FN, false negative hits, FP, false positive hits, GFA, genetic function approximation, H, hydrophobic feature, HBA, hydrogen bond acceptor, HBD, hydrogen bond donor, HIV-1, human immunodeficiency virus 1, KEGG, Kyoto Encyclopedia of Genes and Genomes, MB, metal binding feature, MNA, multilevel neighborhoods of atoms, m. p., melting point, NI, negative ionizable feature, PASS, Prediction of Activity Spectra for Substances, PDB, Protein Databank, PGE_2_, Prostaglandin E_2_, R, ring feature, ROCS, Rapid Overlay of Chemical Structures, OE, overall enrichment, OECS, ROCS OpenEye ComboScore, OEST, ROCS OpenEye shape Tanimoto, SEA, Similarity Ensemble Approach, SERMs, selective estrogen receptor modulators, Tc, Tanimoto coefficient, TN, true negative hits, TP, true positive hits, WOMBAT, World of Molecular Bioactivity, XVOL, exclusion volume

## Abstract

Computational methods can be applied in drug development for the identification of novel lead candidates, but also for the prediction of pharmacokinetic properties and potential adverse effects, thereby aiding to prioritize and identify the most promising compounds. In principle, several techniques are available for this purpose, however, which one is the most suitable for a specific research objective still requires further investigation. Within this study, the performance of several programs, representing common virtual screening methods, was compared in a prospective manner. First, we selected top-ranked virtual screening hits from the three methods pharmacophore modeling, shape-based modeling, and docking. For comparison, these hits were then additionally predicted by external pharmacophore- and 2D similarity-based bioactivity profiling tools. Subsequently, the biological activities of the selected hits were assessed in vitro, which allowed for evaluating and comparing the prospective performance of the applied tools. Although all methods performed well, considerable differences were observed concerning hit rates, true positive and true negative hits, and hitlist composition. Our results suggest that a rational selection of the applied method represents a powerful strategy to maximize the success of a research project, tightly linked to its aims. We employed cyclooxygenase as application example, however, the focus of this study lied on highlighting the differences in the virtual screening tool performances and not in the identification of novel COX-inhibitors.

## Highlights

• Selected common virtual screening tools were applied in parallel.
• Top-ranked hits were investigated with bioactivity profiling tools.
• Compounds were tested in vitro for COX activity.
• The prospective performance of all applied programs was evaluated and compared.

## Graphical abstract

**Figure:**
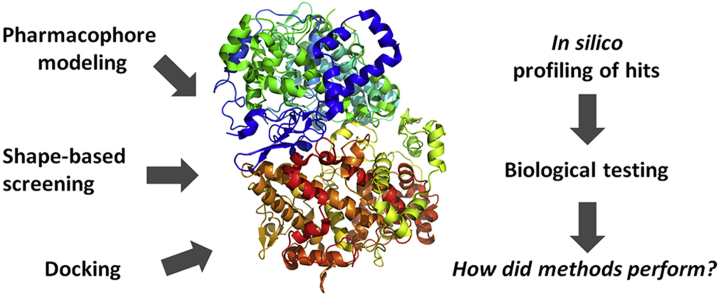

## 1 Introduction

In silico tools are nowadays well integrated in the drug development process and are considered a complementary approach to experimental methods. They have proved to successfully identify ligand–target interactions [1] and to enrich active compounds in the libraries selected for biological testing [2]. Although they were originally used for the prediction of novel disease-modulating compounds for a specific target, additional strategies arise [1]. Besides classical lead-identification, in silico tools can also be deployed for the investigation of off-target interactions. These additional interaction predictions can comprise basically all targets for which sufficient data, structural or ligand-based, is available for model building. *Via* the parallel screening of multiple targets against one compound, so-called bioactivity profiles can be generated. They can help to predict adverse events as well as pharmacokinetic properties [3–6] and may aid to prioritize and identify the most promising drug candidates and to exclude compounds with a bad risk profile [7]. In principle, a lot of different approaches can be used to address these issues. Among the most commonly used in silico tools are docking and similarity-based methods. Similarity-based methods rely upon the assumption that similar molecules exert similar biological effects. Compounds can be compared according to their 2D structure (2D similiarity-based methods), or, in a 3D approach, according to their size and shape (shape-based modeling) or their electrochemical features (pharmacophore modeling). According to IUPAC, “a pharmacophore is the ensemble of steric and electronic features that is necessary to ensure the optimal supra-molecular interactions with a specific biological target structure and to trigger (or to block) its biological response” [8]. These features represent properties like hydrogen bond donor (HBD)/acceptor (HBA) or hydrophobic (H) parts of a molecule rather than specific functional groups. In addition, a model can contain exclusion volumes (XVOLs) that mimic the binding site and into which a molecule is not allowed to protrude in order to avoid steric clashes with the target. Shape-based methods, for example Rapid Overlay of Chemical Structures (ROCS) [9,10], can be optimized by also including chemical information in addition to shape characteristics [11]. The main prerequisite for docking is 3D structural information about the target derived from e.g. X-ray crystallography, NMR studies, or homology modeling. Basically, docking comprises two steps. First, the ligand is fitted into the binding site, and second the “quality” of the interaction pose is evaluated with scoring functions. The results can then be ranked according to their scores with compounds more likely to be active ranked at the top [12].

Within this study, we applied established virtual screening tools based on all methods mentioned above in parallel and investigated their performances in a prospective screening. In principle, multiple virtual screening software tools are available for every method.

For our pharmacophore-based investigations, we selected the program LigandScout [13]. In a recent comparative study of pharmacophore-based virtual screening programs LigandScout was within the best performing tools for all applied case studies when it comes to early enrichment rates [14]. The software program ROCS was employed for shape-based virtual screening. For the docking studies, we used the program GOLD [15,16], because it was shown to be among the best performing and most robust programs in a comparison of docking tools [17]. Also a recent study highlighted the good performance of GOLD, thereby approving our choice [18].

For additional bioactivity profiling, but not for selecting test compounds, we employed two 2D similarity-based (SEA [19] and PASS [20]) and two external pharmacophore-based (PharmMapper [6] and PharmaDB [21]) software tools. All these tools screen the compound against a plethora of diverse targets and therefore provide a whole in silico bioactivity spectrum rather than predictions against one single target.

All these methods have been applied successfully for the identification of novel bioactive compounds [22–24], but which method is the most suitable for a specific target class or research questions still remains largely elusive. In addition, in a recent study, we could observe substantial differences even between programs that rely upon the same methodology. Both of the two applied pharmacophore modeling software tools, LigandScout and Discovery Studio [25], were able to identify novel bioactive compounds, but there was no overlap in the retrieved hitlists [26]. We therefore assumed that the differences in the performances may be even more pronounced between programs based on different methods such as shape-based screening and docking as applied in this study.

Several performance evaluations have been published recently [10,21,27–31], which points out the arising interest in addressing this question. However, in most cases different datasets and combinations of methods have been applied, thereby limiting a direct comparison of the results. In addition, most of these studies are retrospective, and the performance of the investigated methods may differ in a prospective screening. Therefore, an exhaustive prospective comparison of both ligand- and target-based methods still needs to be performed. Tresadern et al. also included a prospective part in their comparison, where they evaluated the performance of Extended-connectivity fingerprints (ECFP) 6, Ftrees, Topomers, Cresset FieldScreen, and ROCS OpenEye Shape Tanimoto (OEST), OpenEye ComboScore (OECS), and OpenEye electrostatics in identifying ligands for the corticotropin releasing factor 1 receptor. However, the prospective aspect only comprised Topomers, Cresset, OEST, and OECS. Pharmacophore modeling and docking were missing in this study [28]. In 2010, Krüger and Evers retrospectively compared the enrichment factors of several docking protocols, ROCS, Feature Trees, and Scitegic Functional Fingerprints and investigated the hitlist complementarity. For their study, they used four different targets, namely angiotensin-converting enzyme (ACE), thrombin, human immunodeficiency virus 1 (HIV-1) protease, and cyclooxygenase (COX) 2 [30]. We also selected COX-2, in addition to COX-1, for our studies, because a lot of biological data is already available to theoretically set up the models. Although we used both isoforms for the experiment, we have to clarify at the beginning that we did not intend to investigate the mechanisms underlying COX-selectivity. We employed COX only as application example, the identification of novel COX-inhibitors is therefore also of subordinate importance. The aim of the study is the investigation of the performances of several different software tools for the identification of inhibitors for classical enzymes and to determine their advantages and limits. This study does not provide a quantitative evaluation of the programs, but highlights the observed differences resulting from the distinct virtual screening concepts. For this purpose, we selected widely used, representative virtual screening programs (LigandScout for pharmacophore modeling, ROCS for shape-based modeling, and GOLD for docking) and bioactivity profiling tools (SEA and PASS for 2D-similarity based profiling and PharmaDB and PharmMapper for pharmacophore-based profiling). They were applied independently for the prediction of COX activity of selected virtual hits. The biological testing of the hits allowed for a prospective evaluation of all applied tools and their direct performance comparison.

## 2 Methods

### 2.1 Study design

**Fig. 1:**
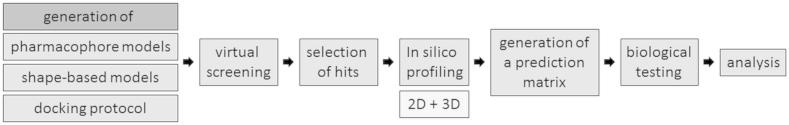
Study design.

Over the course of this study, several shape-based models as well as a docking protocol were developed and theoretically validated. In addition, a previously reported collection of already validated (theoretically and experimentally) COX pharmacophore models created with LigandScout [26] was included in the study. This served as a direct comparison of the performances of these virtual screening tools and was intended also as a follow-up study to the previously published comparison of the two pharmacophore-based virtual screening tools Discovery Studio and LigandScout [26]. In this study, we could observe quite substantial differences in the performances of these two programs [26], and assumed that the differences might be even more pronounced when using methods underlying a different virtual screening concept, such as shape-based modeling or docking. The best performing pharmacophore and shape-based models as well as the docking workflow were used for virtual screening of the in-house database from the Institute of Pharmacy at the University of Innsbruck. The activity of top-ranked hits was also predicted with additional external in silico profiling tools, which were applied in parallel as independent classification (active/inactive) software. All predictions of every program were summarized in a prediction matrix (Table 4). After the biological testing, the hitlists were analyzed and the performances of the applied tools were evaluated and compared (Fig. 1).

### 2.2 Hardware specification

All processes and predictions were performed on a multi-core workstation with 2.4+ GHz, 8 GB of RAM, a 1+ TB fast mass storage, and a NVIDIA graphical processing unit. All programs run on the Windows 7 platform, except GOLD 5.0.1, which was installed on openSUSE 12.2.

### 2.3 Screening database

The in-house database of the Institute of Pharmacy at the University of Innsbruck, Austria, was used for virtual screening. This virtual database merges the chemical structures of compounds synthesized at the Pharmaceutical Chemistry, isolated from plants at the Pharmacognosy, stored at the Pharmaceutical Technology, or used for student training in analytics. It contains already approved drugs as well as published molecules with unknown biological effects and completely new substances. In total, 2719 diverse compounds are collected in the in-house database. The structures of the compounds were manually assembled, therefore the database did not undergo further treatment such as changes in the protonation state.

Since every software program requires a different file format, please refer to the respective method for a detailed description of the database preparation.

### 2.4 Prospective screening methods

#### 2.4.1 Pharmacophore modeling

The program LigandScout [13] version 3.02 was employed for the generation of our internal pharmacophore models and the prospective pharmacophore-based virtual screening. LigandScout facilitates both structure- and ligand-based modeling: In a structure-based approach, the experimentally determined interaction patterns are extracted from ligand–target complexes such as X-ray crystal structures. When using a ligand-based approach, conformational models of known active ligands are aligned to identify common electrochemical features. For further optimization of initial pharmacophore models derived from both approaches, pharmacophore features can be manually added and deleted, and their size and feature weight can be adapted. In addition, optional features can be defined. These features can, but don't have to be mapped, however, if these features are matched the score increases compared to compounds that don't fit them. As default, interaction patterns are represented by HBD, HBA, aromatic (Ar), positively (PI) and negatively (NI) ionizable, metal binding (MB), and H features. Additionally, steric constraints can be included in a model to mimic the binding pocket and prevent mapping of compounds that would clash with the binding site. Over the course of a virtual screening, LigandScout maps a pre-calculated library of conformations to the pharmacophore model, calculates how many features a database compound matches and the overlap of the feature centers [33].

For a detailed description of the COX pharmacophore model generation and validation, please refer to Temml et al. [26]. Multiple COX–inhibitor complexes are available in the Protein Databank (PDB) [32], therefore we exclusively employed structure-based approaches for pharmacophore model generation. Initial pharmacophore models were automatically created for all complexes deposited in the PDB [32] as described above. The models were manually refined with an active and decoy set, containing 100 and 99 active compounds for COX-1 and COX-2, respectively, and 4984 decoy molecules collected from the ChEMBL database [34]. The smiles codes of these compounds are provided in the supporting information. For the prospective screening, a maximum number of 100 conformers was calculated for every compound in the in-house database using OMEGA [35–37] implemented in LigandScout [13]. We used models generated with LigandScout as described previously [26] for the prospective screening of the in-house database. These models and their hitlists were then further analyzed.

#### 2.4.2 Shape-based modeling

ROCS [9,10] employs a Gaussian function to compare the shape of a query molecule used for model building and the shapes of screening compounds present in a database. The degree of Gaussian function overlap is calculated as Shape Tanimoto score [38], analogous to the Tanimoto coefficient (Tc) used for 2D similarity-based investigations, and it also ranks from zero to one. ROCS also allows for the addition of so-called color features, which, like pharmacophore features, represent electrochemical feature types like HBD, HBA, anion (A), cation (C), ring (R), and H moieties. Mapping of the database compounds to the color features is measured with the ScaledColor score, where the individual fit values are normalized to the score of the query molecule (which retrieves the maximum score). The ScaledColor score also ranks from zero to one. Both fit scores are combined in the ComboScore *via* simple addition, thereby equally weighting both the shape overlap and the mapping of the features. Consequently, the ComboScore ranks from zero to two.

Within this study, vROCS version 3.0.0 [9,10] was used. In total, 33 different shape-based models were generated using 23 different PDB-entries. For the virtual screening, however, only two models based on the crystal complexes of COX-1 and celecoxib (PDB-entry 3KK6 [39]) and methyl ester flurbiprofen (PDB-entry 1HT5 [40]) were selected, because they performed best during the theoretical validation.

For the theoretical validation of all ROCS-models, the data-sets from the pharmacophore model generation were used, which are briefly described above [26]. Since we did not discriminate between the two isoforms, the files for COX-1 and -2 actives were merged and 13 duplicate molecules were removed (the smiles codes of the data-set compounds are provided in the supporting information). The sd-files were converted to oeb.gz-files using the default settings of OMEGA 2.3.2 [35–37], thereby generating a maximum number of 200 conformers per molecule. For every structure in the in-house database, one input conformation was calculated with CORINA [41] and this database was again converted to an oeb.gz-file with the default settings of OMEGA 2.3.2. Virtual screening was performed with default settings and the results were ranked according to the ComboScore.

#### 2.4.3 Docking

The software program GOLD 5.0.1 [15,16] was used for the molecular docking of compounds into the crystal structure of COX-1 complexed with methyl ester flurbiprofen (PDB entry 1HT5 [40]). GOLD is based on a genetic algorithm, which mimics evolutionary selection processes such as crossover and mutation. In this context, the chromosomes represent possible ligand binding modes within the binding pocket and consist of two binary and two integer strings, where the binary strings encode the conformations of the ligand and the protein, and the integer strings the formation of hydrogen bonds of both the ligand and the protein and the ligand with itself.

The fitness function applied in this study, the GoldScore, is composed of the hydrogen bond energy, the steric energy upon binding of the ligand, and the internal energy of the ligand alone [16].

For protein preparation, 9402 hydrogens were added, all waters (150) were deleted, and the ligand was extracted. The area of 6 Å surrounding the ligand was defined as binding site. The docking was performed in virtual screening mode and the GoldScore was selected as Fitness function. The output was restricted to ten poses per compound. For every structure in the in-house database, one conformation was calculated with CORINA [41]. Only one crystal structure of a COX–ligand complex was employed for docking, because it was reported in the literature that the binding site does not undergo conformational changes upon binding of different inhibitors [40].

### 2.5 Retrospective profiling tools

The 20 best-ranked hits of the pharmacophore-based, the shape-based, and docking-based virtual screenings were merged to a combined hitlist, which was further investigated with external pharmacophore- and 2D similarity-based profiling tools.

#### 2.5.1 Pharmacophore-based methods

*PharmaDB*. Since version 3.5, Discovery Studio includes a collection of 68,056 structure-based pharmacophore models for 2556 different targets [21]. They were automatically generated from 7687 protein–ligand complexes from the PDB, and subsequently selected based on a genetic function approximation (GFA) model estimating the restrictivity of the pharmacophore models. Every included model consists of three to six features. A maximum number of 10 models was created for every complex. The models were combined in a database called PharmaDB, which can be used for compound profiling. The merged hitlist was screened by the most selective pharmacophore model created with every COX–ligand complex. This subset of pharmacophore models is provided in Discovery Studio and can be selected during setting up a screening run. The selectivity of the models was determined by using the same GFA model as described above [21].

*PharmMapper*. PharmMapper [6] is a freely accessible online pharmacophore collection (http://59.78.96.61/PharmMapper/) intended for compound profiling. It consists of more than 7000 structure-based models for 1627 targets. The pharmacophore models were automatically generated with LigandScout [13] and then manually analyzed, however, a distinct algorithm is used for virtual screening [6]. Every compound of the merged hitlist was uploaded to the PharmMapper server separately and a maximum number of 300 conformers was generated. All targets in the pharmacophore collection were selected for profiling, however, the output was limited to the 300 best-ranked models, and only models with at least six features were counted as hits.

#### 2.5.2 2D-similarity-based methods

*SEA* [19] (http://sea.bkslab.org/) and PASS [20] (http://www.pharmaexpert.ru/passonline/) are 2D-similarity-based profiling tools that are freely accessible online. SEA uses ECFP4 fingerprints to describe the 2D structure of compounds, and the similarity between single pairs of compounds is calculated as Tc. SEA employs a statistical model to correct for “random similarity” to optimize the results [19]. It is based on structures of ligands with annotated biological activities from the ChEMBL Medicinal Chemistry Database, the MDL Drug Data Report, the World of Molecular Bioactivity (WOMBAT) database, and the Kyoto Encyclopedia of Genes and Genomes (KEGG) database to compare the structure and predict the activity of query molecules. The smiles code of every compound from the merged hitlist served as input, and within this study the ChEMBL version 10 and the WOMBAT database were selected for profiling.

*PASS* relies on the same principle, but applies the Multilevel Neighborhoods of Atoms (MNA) descriptor for representation. It predicts the probability of a compound x to be active against target y with an improved naïve Bayes algorithm. The online version predicts over 3500 targets and biological activities, which were collected manually from literature [20]. All molecules were uploaded as mol-files.

### 2.6 Experimental testing

#### 2.6.1 Materials

All compounds except fosfestrol were obtained from our in-house library. A free sample of fosfestrol was a kindly provided by Aronis (Moscow, Russia, http://www.aronis.ru/). Before biological testing, the melting points (m. p.) of all compounds were measured with a Kofler heating bench (Wagner & Munz, Type WME) to confirm their identity. The heating bench was “calibrated” before use with benzil (m. p. of 95 °C), acetanilide (m. p. 114.5 °C), phenacetin (m. p. 134.5 °C), benzanilide (m. p. 163 °C), salophen (m. p. 191 °C), dicyandiamide (m. p. 201 °C), and saccharin (m. p. 228 °C). In case the melting point differed from the literature data, the compounds were additionally analyzed with ^1^H NMR or HPLC mass spectrometry.

#### 2.6.2 Biological testing

Selected compounds were tested for their COX-inhibiting activity as described earlier [42]. All compounds and the positive control (*S*)-ibuprofen (Sigma Aldrich) were dissolved in DMSO (Sigma Aldrich) and tested in triplicates at a concentration of 20 μM. Briefly, 5 μl of ram seminal vesicle COX-1 (1 U) (Sigma–Aldrich) or human recombinant COX-2 (0.5 U) (Sigma–Aldrich) were added to 180 μl of cofactor mix containing 5 μM hematin (Sigma–Aldrich), 18 mM l-epinephrine (Sigma–Aldrich), and 50 μM Na_2_EDTA (Roth) in 100 mM Tris-buffer (pH 8.0) (Biorad). After addition of 10 μl test compound, (*S*)-ibuprofen, or DMSO, the reaction mix was incubated for five minutes at room temperature. The reaction was started by adding 5 μl of 10 μM arachidonic acid (AA) (Sigma–Aldrich) solved in ethanol. After incubation at 37 °C for 20 min, the reaction was stopped with 10 μl 10% formic acid and the samples were diluted 1:15 in assay buffer. Prostaglandin E_2_ (PGE_2_) concentration was determined using the EIA Kit produced by Enzo Life Sciences according to the manufacturer's protocol. Absorbance was measured with a Tecan Infinite M200 (Tecan Group) plate reader at 405 nM. Results of test compounds and positive control were calculated as % inhibition compared to untreated samples. IC_50_-values were measured for all compounds that exhibited an inhibition of approx. 50% at 20 μM concentration. The IC_50_ values were calculated as average of at least two independent experiments performed at various concentrations and in triplicates.

### 2.7 Analysis of the results

After the biological testing, the results were analyzed within three categories. The first category, early enrichment (EE), investigated to which percentage the predicted activity of the top-20 ranked molecules could also be confirmed in the biological assay.

(1) $$EE=\left(\frac{number\;of\;active\;compounds\;in\;the\;top-20\;hitlist}{number\;of\;all\;compounds\;in\;the\;top-20\;hitlist}\right)\times 100$$

The second category, overall enrichment (OE), analyzed if these compounds were also predicted by the other methods above a certain activity cut-off. The cut-offs were defined either according to the validation cut-off or to published data. For ROCS, a cut-off of a ComboScore ≥1.00 was taken according to the theoretical validation cut-off, which is described later in more detail. For SEA, the cut-off was set at an E-value ≤−4 as proposed by Lounkine et al. [5]. The cut-off was set at Pa ≥0.5 for PASS, because it was shown that most active molecules are distributed above that threshold [43]. No cut-off was used for the pharmacophore modeling approaches. The cut-off for docking was determined as a GoldScore of ≥40.0 in the theoretical validation.

(2) $$OE=\left(\frac{number\;of\;active\;compounds}{number\;of\;predicted\;compounds\;from\;the\;merged\;hitlist}\right)\times 100$$

The last category investigated the predictive power of the different tools and for this purpose, the numbers of true positive (percentage of predicted and biologically active compounds, TP), false positive (percentage of predicted but biologically inactive compounds, FP), true negative (percentage of compounds that were not predicted and indeed were biologically inactive, TN), and false negative (percentage of compounds that were not predicted but were biologically active, FN) hits, and the percentages of overall true predictions (in the latter referred to as accuracy (Acc [44])) were calculated. A prediction was considered as true, when a predicted molecule was active in the biological testing, but also, if it was not predicted as a hit and inactive in the assay. The same cut-offs as described above were applied.

(3) $$Acc=\left(\frac{TP\;+\;TN}{TP\;+\;TN\;+\;FP\;+FN\;}\right)\times 100$$

To further analyze the composition of the top-20 hitlists, diversity metrics were calculated with Discovery Studio 3.5 [25]. AlogP, molecular weight, number of HBD and HBA, number of rotatable bonds, number of rings in general and aromatic rings in particular, and the molecular fractional polar surface area were selected as parameters. For calculation of 2D structural similarity distances, ECFP4 Fingerprints were used and the similarity was determined with the Tc.

## 3 Results

### 3.1 Prospective screening methods

#### 3.1.1 Pharmacophore modeling

**Fig. 2:**
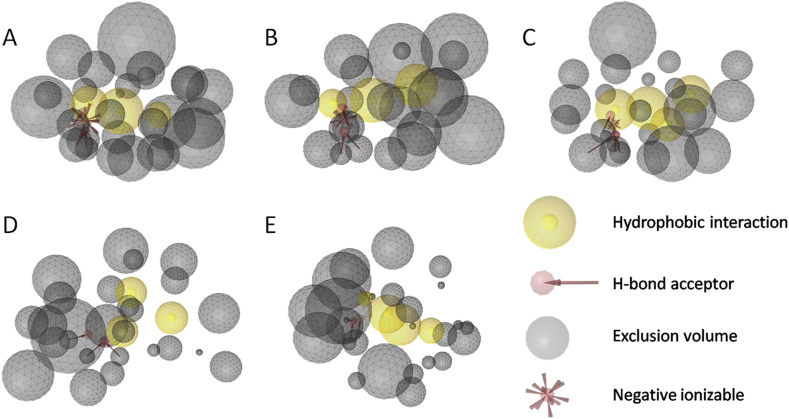
Pharmacophore models for COX-1 (A–C) and -2 (D–E). (A) The model 1EQG consists of three H features, three HBAs, one NI feature, and 27 XVOLs. (B) The model 1Q4G contains three H features, of which the smallest one is optional, three HBAs, one NI feature, and 22 XVOLs. (C) In addition to 24 XVOLs, model 2AYL contains four H, four HBA, and one NI feature. (D) The COX-2 model 3LN0 is composed of three H and three HBA features and 26 XVOLs. (E) The model 3NTB consists of 4 H features, of which the smallest one is again optional, three HBAs, and 26 XVOLs.

**Fig. 3:**
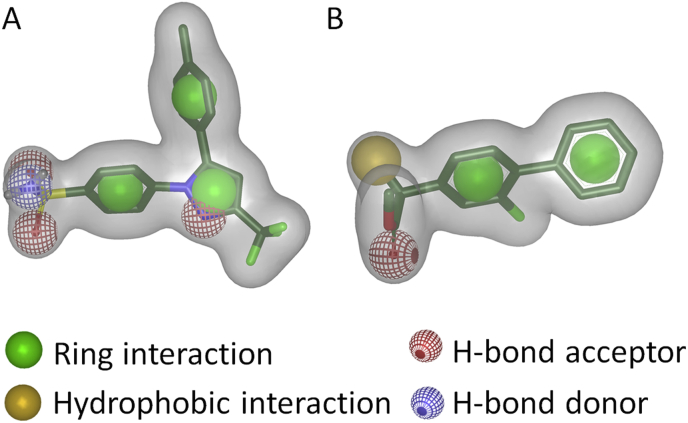
ROCS models generated with celecoxib (A) and methyl ester flurbiprofen (B). (A) In addition to the shape, the celecoxib comprised of three R features, three HBAs, and one HBD feature. (B) The methyl ester flurbiprofen model contained two R features, one HBA feature, and one H feature.

The generation of the pharmacophore models was described recently [26]. Briefly, pharmacophore models were generated with all suitable PDB entries available at that time, leading to 37 COX-1 and 27 COX-2 models. Many of these models mapped redundant molecules from the test set, and therefore only a selection of the best-performing, complementary models was applied for the prospective part [26]. For the virtual screening of the in-house database, the COX-1 models 1EQG (Fig. 2A), 1Q4G (Fig. 2B), 2AYL (Fig. 2C), and the COX-2 models 3LN0 (Fig. 2D) and 3NTB (Fig. 2E) were used. Those models were selected, because they retrieved the compounds with the highest relative pharmacophore fit value in the course of the prospective screening. The three COX-1 models were generated with the PDB entries 1EQG [40], 1Q4G [45], and 2AYL [46], in which COX-1 forms a complex with the inhibitors ibuprofen, α-methyl-4-biphenylacetic acid, and flurbiprofen, respectively. The crystal structures of the two inhibitors SD-8381 (PDB entry 3LN0 [47]) and 6-methylthionaproxen (PDB entry 3NTB [48]) provided the basis for the two COX-2 pharmacophore models, respectively. All hits derived from these models were merged and ranked according to their relative geometric fit values, and the 20 best-ranked hits were selected for biological testing (Table 1).

#### 3.1.2 Shape-based modeling

Several different shape-based models were generated with ROCS [9,10]. All the models generated in the course of this study were based on co-crystallized COX–ligands, because they represent the bioactive conformations. The models were validated with the same active and decoy set as was used for the pharmacophore models. In total 33 different models covering 23 PDB-entries were generated and two of them were finally selected for virtual screening. Together, they scored 95% of the compounds from the actives set, including both COX-1 and COX-2 active compounds, with a ComboScore ≥1.0 and the AUCs in the ROC plots (for a detailed description of ROC plots please refer to Triballeau et al. [49]) were 0.71 and 0.70, respectively. These two models were generated with the co-crystallized ligands of the PDB-entries 3KK6 [39] (celecoxib bound to COX-1, Fig. 3A) and 1HT5 [40] (methyl ester flurbiprofen bound to COX-1, Fig. 3B) using the default settings. Although several steps were undertaken to improve the automatically generated models manually, the unmodified ones performed best. The two models were then used for virtual screening the in-house database. The results were ranked according to the ComboScore. The 20 highest scored hits were selected for biological testing (Table 2).

#### 3.1.3 Docking

**Fig. 4:**
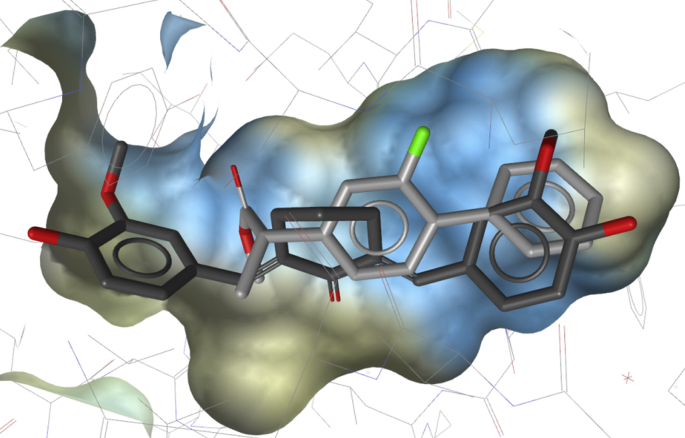
Highest scored docking pose of cyqualon (**3**) (dark gray) and the co-crystallized ligand methyl ester flurbiprofen (light gray).

For the docking, we aimed to use the X-ray crystal structures used for shape-based modeling too. However, docking into the PDB-entry 3KK6 [39] could not be successfully validated. Next, the crystal structure of methyl ester flurbiprofen bound to COX-1 was evaluated for the docking studies (PDB-entry 1HT5 [40]). For validation of the docking workflow, the co-crystallized ligand was re-docked into the binding site with an RMSD-value of 1.029 Å. In addition, a subset of 50 active compounds and 60 decoys from the test set was docked. The number of test set compounds was restricted due to time-reasons; however, we focused on diverse molecules. The diversity of the subset was analyzed with Discovery Studio's “Calculate Diversity Metrics” using the ECFP4 fingerprints and the Tc. The active compounds and decoys retrieved an average fingerprint distance of 0.894 and 0.869, respectively. Based on the docking run, we selected an activity cut-off of GoldScore ≥40.0, because 41 of the 50 active compounds (82.0%), but only 15 of the decoys (25.0%) were ranked above this threshold. This led to an AUC of 0.76. In the prospective screening, the in-house database was docked into the binding site. The results were ranked according to the GoldScore, and the 20 best-ranked hits were selected for biological testing (Table 3). The highest ranked docking pose of the top-20 ranked compound cyqualon (**3**) is depicted in Fig. 4.

#### 3.1.4 Investigation of the merged hitlist

All the compounds predicted with the three approaches mentioned above were merged to one hitlist, which contained 52 molecules after the removal of 7 redundant compounds (picosulfate was predicted by all three methods). Subsequently, the hitlists of pharmacophore modeling, shape-based screening, and docking were analyzed to investigate, whether some of the remaining compounds of the merged hitlist were predicted above the defined cut-off, indicating consensus hits. After this merger, pharmacophore modeling predicted 25 of the 52 compounds, docking indicated 46 hits, and shape-based screening estimated all 52 compounds as active substances, respectively. In addition, all 52 compounds were profiled with the external pharmacophore-based profiling tools PharmaDB and PharmMapper as well as with the 2D similarity-based methods SEA and PASS.

### 3.2 Retrospective profiling tools

#### 3.2.1 Pharmacophore-based methods

*PharmaDB*. Only the 14 most selective models for COX with and without shape included in the PharmaDB were selected for the screening to minimize the screening time and avoid large data volumes. 44 of the 52 molecules mapped at least one pharmacophore model, and seven pharmacophore models were mapped in total. The highest fit values for all compounds that mapped at least one model are listed in Table 4.

*PharmMapper*. The PharmMapper profiling predicted 11 compounds to interact with either COX-1 or COX-2 within the top-300 ranked targets (Table 4).

#### 3.2.2 2D-similarity-based methods

*SEA*. In the course of the SEA profiling, 17 molecules were predicted with an E-value ≤−4 and therefore considered as hits (Table 4).

*PASS*. In the profiling with PASS, all results with a Pa-value ≥ 0.5 were considered as hits, in this case 24 out of 52 compounds (Table 4).

### 3.3 Generation of a prediction matrix

The predictions generated with pharmacophore-based and shape-based screening and molecular docking for the 52 unique compounds from the merged hitlist were combined in a prediction matrix. In parallel, the 52 compounds were investigated with the bioactivity profiling tools SEA, PASS, PharmaDB, and PharmMapper, and their predictions were added to the matrix. In case COX-activity was reported, data from the literature was included as well. All predictions are summarized in Table 4.

### 3.4 Preliminary analysis of the merged hitlist

For 14 of the proposed compounds, experimental data was already available in the scientific literature. This was the case for alclofenac [40], berberine [50], carprofen [51], (S)-ibuprofen [61], fenbufen [52], flunixin meglumine [53], (*R*)-ibuprofen [60], indometacin [54], indoprofen [55], ketoprofen [56], meclofenamic acid [57], mefenamic acid [58], pirprofen [59], and tiaprofenic acid [55]. All these compounds, except berberine were found to inhibit COX. Berberine modulates the AA cascade, and PGE_2_-levels are decreased after berberine treatment, but this effect is regulated on the transcriptional level, not by direct inhibition of the enzyme [50]. (*R*)-ibuprofen was well accepted as the inactive stereoisomer of racemic ibuprofen. However, Duggan et al. reported that (*R*)-ibuprofen-mediated COX-inhibition is substrate-selective. While PGE_2_ formation from AA is only inhibited at high concentrations, the oxidation of endocannabinoids is prevented with an IC_50_ of approximately 10 μM. The crystallization of other (*R*)-profens revealed the same binding site and an analogous binding mode compared to the (*S*)-enantiomers. Apparently, also (*R*)-profens bind to the catalytic center of COX, and therefore (*R*)-ibuprofen was considered as active [60]. Evidence for COX-inhibition was given for sulfasalazin, however, no IC_50_ was available and the present data suggest a very high value [62]. Unfortunately, the purity and identity of sulfasalazin could not be confirmed in our analyses, thereby making it impossible for us to experimentally test this compound. Due to the ambiguous data that exists, we could not define the biological activity of sulfasalazine and excluded it from the final analysis list. In the case of cyqualon (**3**), COX-activity was published by one group [63]. However, the investigation was only qualitative and no IC_50_-value was determined. In contrast, Tham et al. reported a study, where cyqualon (**3**) did not prevent PGE_2_-formation in stimulated differentiated U937 cells [64]. To finally determine the biological activity of cyqualon (**3**) and measure the IC_50_ value, cyqualon (**3**) was included in the experimental testing. The identity of 4-deoxypyridoxine-5-phosphate, flavoxate hydrochloride, sulfasalazine, and triflocin could not be confirmed by melting point and NMR analyses, so they were excluded from biological testing. Two further compounds, dormin and sulfamidopyrine, were not available anymore. Finally, 32 compounds were subjected to experimental testing.

### 3.5 Biological testing

**Fig. 5:**
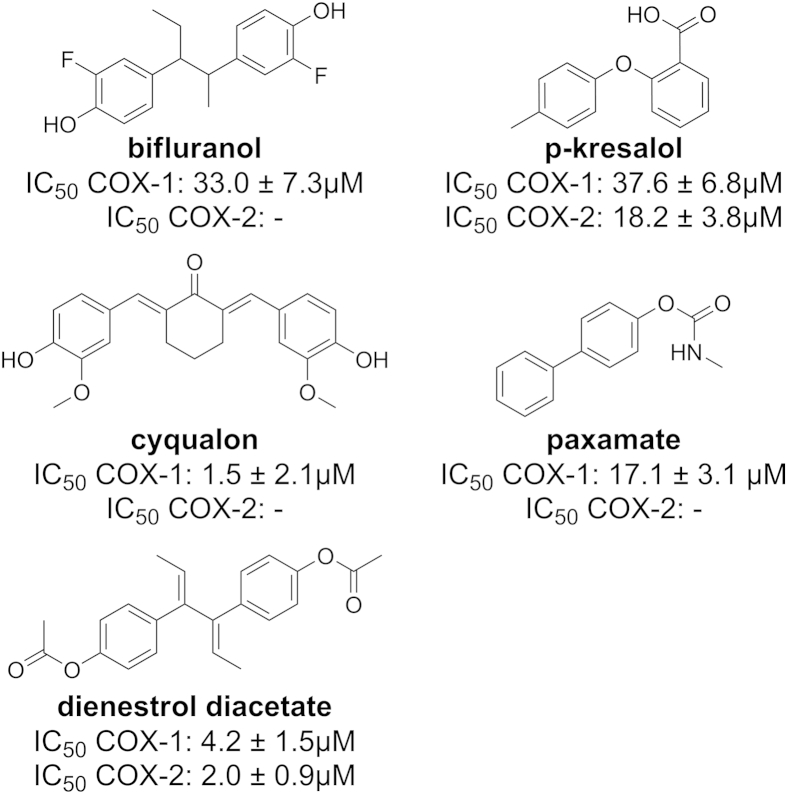
Structures and IC_50_ values of the active compounds.

In the biological assay, 5 of the 32 tested compounds were active with IC_50_ values between 2 and 38 μM. These compounds were bifluranol (**1**), dienestrol diacetate (**5**), cyqualon (**3**), p-kresalol (**2**), and paxamate (**4**). Interestingly, bifluranol (**1**), cyqualon (**3**), and paxamate (**4**) were COX-1 selective, while dienestrol diactetate (**5**) and p-kresalol (**2**) inhibited both isoforms. This is surprising, because bifluranol (**1**) and dienestrol diacetate (**5**) share the same scaffold and differ only in the existence of the double-bonds and the substitutions on the phenyl-moieties. COX-2 is assumed to have a larger binding pocket, however, in this case the smaller compound (bifluranol (**1**)) appears to be COX-1 selective, while the larger one (dienestrol diacetate (**5**)) inhibits both. IC_50_ values and chemical structures of all active compounds are displayed in Fig. 5 and Table 5, respectively. For a detailed list of the structures and biological activities of the inactive compounds, please refer to the supporting information.

### 3.6 Evaluation of the prospective screening methods

#### 3.6.1 Pharmacophore modeling

The application of our own pharmacophore models appeared to be very successful in general and clearly outperformed the other methods in the EE. From the top-ranked compounds 14 out of 18 predicted molecules (dormin was not available and the identity of triflocin could not be confirmed) were found to be active, leading to an EE hit rate of 77.8%. The hit rate of OE was slightly lower and the Acc even higher, with 72.7% and 82.6%, respectively. The overall hitlist consisted of 34.8% TP, 47.8% TN, 13.0% FP, and 4.4% FN hits. The EE hitlist of pharmacophore-based screening, if one considers only the true actives, contained a lot of well-established COX-inhibitors (approximately 60% of the predicted compounds). This seems problematic for a true prospective experiment, however, all these compounds were not included in the test- and trainings set and were therefore considered “real” hits. The successful filtering of active compounds from the database showed the advantages of the method and suggested the identification of novel compounds in the 20 highest ranked molecules also if another database was screened. This is supported by the fact, that bifluranol (**1**), dienestrol diacetate (**5**), and p-kresalol (**2**), were identified as novel COX-inhibitors. These compounds were only found in the top-20 ranked molecules in the hitlist of pharmacophore-based screening and would not have been tested, if it this method had not been applied.

#### 3.6.2 Shape-based modeling

Biological data for 19 of the top-20 ranked compounds were available, because sulfamidopyrine was not available for testing anymore. The activity of 9 of these 19 compounds could be confirmed, resulting in an EE hit rate of 47.4%. This is clearly better that the performance in the OE and the Acc category, where the hit rate was 39.1% in both categories. The Acc value was not higher, because all compounds in the merged hitlist were predicted, and therefore no TN, and consequently also 0.0% FN were found. Approximately 61% of hits were FPs, and the remaining 39.1% were TP hits. Similar to pharmacophore modeling, also the top-20 hitlist of shape-based virtual screening contained a large amount of well-known COX-inhibitors (approximately 40% of the predicted compounds). In addition to the known ligands, the shape-based screening allowed for the identification of another new COX-inhibitor, namely paxamate (**4**). This compound was only ranked that high by shape-based screening and would have been missed, if this method had not been applied. Shape-based virtual screening yielded approximately 50% EE rate and, together with PharmaDB, obtained the highest TP rate.

#### 3.6.3 Docking

Docking clearly performed worst in EE, with only one active out of 17 predicted compounds (the identity of 4-deoxypyridoxine-5-phosphate, sulfasalazine, and flavoxate hydrochloride could not be confirmed) and a hit rate of 5.9%. This is not surprising, since the GoldScore was the only criteria for the selection of compounds. The results were not inspected manually to avoid bias, and the limits of scoring in docking are a well-known problem [12,65]. However, docking performed surprisingly well in OE with 17 out of 40 predicted compounds being active and a hit rate of 42.5%. The performance even improved when regarding the Acc. 22 of the 46 predictions were correct and lead to 47.8% of true predictions. This formidable performance is, at least in parts, due to the narrow cut-off used in the study, which improved the classification of inactive compounds. In detail, docking retrieved 10.9% TN, and only 2.2% FN hits. The remaining hits were either correctly (37.0%) or incorrectly (50.0%) classified as active. The new active compound in the top-20 hitlist of docking, cyqualon (**3**), is distinct from the common COX-inhibitors considering its structure. It has to be highlighted, that although the EE rate was not good, the new active compound identified with docking represents a completely new scaffold. This molecule was again only ranked so high by docking, and, similar to the other methods, would have been missed if docking had not been applied.

### 3.7 Evaluation of the retrospective profiling tools

#### 3.7.1 Pharmacophore-based methods

For the two pharmacophore-based profiling tools PharmMapper and PharmaDB, no data is available for the EE category. Every single compound has to be profiled separately in PharmMapper, thereby making it almost impossible to apply it for large databases. In principle, it is possible to screen large databases with the PharmaDB, but this is very time-consuming and produces a lot of data. So we restricted our studies for all profiling tools to OE and Acc.

*PharmaDB* performed worse than PharmMapper, but still surprisingly well if one considers that all the models were generated using an automated protocol. In OE, 18 out of 39 (hit rate of 46.2%) predicted compounds were active, and concerning Acc, 18 out of 46 (hit rate of 54.4%) predictions were true. In silico profiling with PharmaDB retrieved 39.1% TP, 15.2% TN, 45.7% FP, and 0.0% FN hits.

*PharmMapper* The biological activity of 11 out of 19 compounds predicted by PharmMapper could be confirmed, leading to a hit rate of 57.9% in OE. The performance in the Acc category was even better, with 31 out of 46 predictions being true and a hit rate of 67.4%. In total, the hitlist of PharmMapper consisted of 23.9% TP, 43.5% TN, 17.4% FP, and 15.2% FN hits.

#### 3.7.2 2D-similarity-based methods

Analogous to PharmMapper, every compound has to be profiled separately with SEA and PASS and therefore only the molecules of the merged hitlist were investigated.

*SEA* outperformed all other methods in the OE category with 14 active compounds out of 17 predicted ones and a hit rate of 82.4%. Also concerning Acc it performed very well (39 true predictions out of 46, 84.8%). The hitlist of SEA comprised 30.4% TP, 54.4% TN, 6.5% FP, and 8.7% FN hits.

**Fig. 6:**
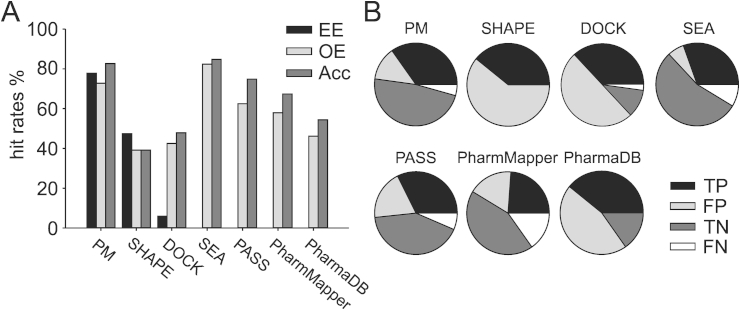
Analyses of the performances. (A) hit rates in % for the categories EE, OE, and Acc. The EE of the retrospective bioactivity profiling tools was not available, because they were not used for prospective virtual screening. (B) Fractions of TP, FP, TN, and FN compounds in the hitlists of the respective tool. PM, pharmacophore modeling; SHAPE, shape-based screening; DOCK, docking.

*PASS* performed quite well, although worse than pharmacophore modeling and SEA, but clearly better than the other methods. In OE, the biological activity of 15 out of 24 predicted compounds could be confirmed, thereby leading to a hit rate of 62.5%. The performance improved in the Acc category, where 34 of the 46 predictions were correct (hit rate 74.8%). In total, PASS yielded 32.6% TP, 42.2% TN, 19.6% FP, and 6.5% FN hits. A detailed graphical representation of all hitrates and calculated metrics is depicted in Fig. 6.

## 4 Discussion

In general, all methods proved to be suitable for the retrospective and prospective identification of COX–ligands, and all performances, except EE for docking, led to hit rates of nearly 40% and more. However, we have to state that the database we used was unintendedly biased. We selected a database that also contained already approved drugs with the intention to, besides the identification of novel COX-inhibitors, also rationalize adverse events that had been observed with the administration of a certain medication. Since many anti-inflammatory drugs are already known, this led to an enrichment of COX-inhibitors *per se*. However, none of these compounds was included in the test- or trainings set for setting up the models. Therefore, all of them can be counted as true hits.

The selection of the virtual screening method strongly depends on the aim of a project and the available resources for biological testing. For the identification of structurally new and diverse ligands, docking seemed to be the most suitable method in our study, although one has to accept a high FP rate, especially when only the highest-ranked molecules are considered and without any further filtering of hits. To further investigate the question of hitlist similarity and exploration of chemical space, the diversity metrics of the top-20 ranked compounds for pharmacophore- and shape-based modeling and docking were calculated with Discovery Studio. The values for fingerprint distances ranged from zero to one, meaning that for compounds with a value of zero there is no distance, e.g. compounds are identical from a 2D structural-point of view. With increasing values compounds are becoming more dissimilar. Interestingly, similar values were obtained for the average fingerprint distances for all three methods. The values were 0.84 for shape-based screening, 0.80 for pharmacophore modeling, and 0.88 for docking. A more detailed investigation of the fingerprint distances revealed that there were, however, differences in the minimum and maximum fingerprint distances observed, ranging from 0.00 and 0.97 for shape-based screening to 0.00 and 0.93 for pharmacophore modeling, and 0.62 and 0.97 for docking. Both shape-based and pharmacophore-based screening hitlists retrieved a minimum number of 0.00, because they ranked both (*R*)- and (*S*)-ibuprofen very high. In contrast, the most similar compounds in the docking hitlists retrieved a minimum distance of 0.62. The hitlists also differed in the diversity of the fingerprint features. This value is defined as the number of different features divided by the number of molecules. Thereby high numbers reflect hitlists with a larger variation of fingerprint properties as hitlists with low numbers. The values retrieved in this study ranged from 14.9 for pharmacophore modeling to 16.55 and 23.25 for shape-based screening and docking, respectively. These results also emphasized the differences of the methods in respect to hitlist diversity.

A high hit rate is much more important than hitlist diversity when it comes to ligand profiling and the prediction of off-target effects or adverse events. Especially the number of FN hits should be as low as possible to identify all potentially harmful compounds. Different from a lead identification project, the number of FP, in contrast, can be larger in this setting. There were two tools, ROCS and PharmaDB, which had high FP hit rates (60.9% and 45.7%, respectively), but both also 0.0% FN hits. In addition, these two programs yielded the highest TP rates (39.1% for both) of all applied methods. Although ROCS predicted every compound in the merged hitlist to be active and seemed to be totally unrestrictive, one has to keep in mind that these compounds were already prioritized and pre-selected by other methods, and don't represent a random selection of molecules. Both programs, ROCS and PharmaDB, identified all active compounds in the dataset, thereby making them particularly useful for the prediction of COX-mediated side effects. Recently, AbdulHameed et al. performed a retrospective multi-target study, where they explored the suitability of ROCS for target-fishing, and for several compounds they successfully identified off-targets that had already been described in literature [4].

Also pharmacophore modeling and 2D-similarity based methods turned out to be suitable for activity profiling. The TP hits bifluranol (**1**) and dienestrol diacetate (**5**) share the same scaffold and both belong to the class of selective estrogen receptor modulators (SERMs). Their chemical structure is distinct from the common COX-inhibitors and this shows the applicability of pharmacophore modeling also for scaffold hopping [66]. Another member of this group, chlorotrianisene, was identified by SEA as COX-inhibitor recently, thereby rationalizing the occurrence of abdominal pain that was observed with chlorotrianisene treatment [5]. These examples again highlight the ability of in silico tools to elucidate and predict adverse events. SEA and PASS emerged also as powerful tools, because the investigation of a single compound of interest is extremely fast and easy to handle. On the other side, the non-commercial versions have the disadvantage that only one molecule after the other can be analyzed, which makes the screening of large databases rather difficult. PharmaDB is in principle intended for profiling of whole databases. To investigate the utility of this tool for the generation of a bioactivity spectrum, we performed a profiling run with cyqualon (**3**). Although we only applied the most selective models without shape (7303 models in total), we obtained 457 matching pharmacophores, which represent 6.3% of the database. So despite only screening with a small part of the overall pharmacophore model collection, we got a quite large number of hits. Therefore, the applicability of this tool may be limited by the (unrealistically) large amount of data. We assume that this can in part be attributed to the fact that the composition of the models was restricted to six features, thereby making even the more selective models rather unselective.

The comparison of the three different pharmacophore tools pointed out the importance and the impact of careful model generation and validation. Our own pharmacophore models, which were generated and validated manually, performed clearly better than PharmMapper and PharmaDB, where models were generated automatically (OE and Acc of 72.7% and 82.6% for our own models versus 57.9% and 67.4% for PharmMapper and 46.2% and 54.4% for PharmaDB), respectively. Also PharmMapper, where the automatically generated models were subsequently manually analyzed [6], performed better than the PharmaDB.

In a recent study, Venkatraman et al. compared the performance of different shape-based and 2D fingerprint –based screening tools using 40 targets of the Directory of Useful Decoys (DUD) [67] dataset, including COX-1 and -2. Similar to our results, they observed a better performance of the 2D-similarity tools, which they attributed to the limitation of 3D shape-based methods to cover the bioactive conformation of query and database molecules [31]. However, this may only partly apply for this study since we used co-crystallized ligands for model generation, which are most likely represented in their bioactive conformation, or a very similar one, in the crystal. Krüger and Evers also compared the performances of different in silico methods for the identification of COX-inhibitors, and in this study ROCS performed best of all methods [30]. Unfortunately, they limited their analysis to ROCS, docking, Scitegic Functional Fingerprints, and Feature Trees, and did not include pharmacophore modeling. ROCS and docking performed almost similar in their study, and we also observed no big differences in the performances of these two approaches regarding OE and Acc. In contrast, their 2D-similarity search was clearly outperformed by ROCS and docking. This may be due to the fact, that they used another descriptor (Functional-Class Fingerprints 4, FCFP4) than the ones applied in PASS (MNA) and SEA (ECFP4). In addition, they only used one ligand as a reference molecule, while PASS had a set of 1376 actives for COX-1, 3370 actives for COX-2, and 4216 actives for COX in general. SEA used 119 active compounds for sheep, 27 for mouse, 135 for rat, and 499 for human COX-1, and 1875 active compounds for human, 151 for rat, 186 for sheep, and 253 for mouse COX-2 in the ChEMBL10. These large data volumes allow for covering a much bigger chemical space for one target and may contribute to the excellent performance we observed. Krüger and Evers also noticed, that despite overlapping results, the hitlists of the distinct methods are highly complementary and that every method contributed on its own to identifying active molecules. Considering hitlist complementarity, seven overlapping hits have been obtained in our study in the top-20 hitlists of all three methods. Six of these redundant hits were already known COX-inhibitors, and the one with unknown biological activity, picosulfate sodium, was inactive. Intriguingly, picosulfate sodium was the only compound that was ranked among the top-20 hits by all three methods. All of the six known COX-inhibitors were predicted by both pharmacophore modeling and shape-based screening. In contrast, all of the newly identified COX-inhibitors have only been ranked in the top-20 of one method (a comprehensive discussion of these novel COX-inhibitors is provided in the supporting information). In detail, this comprises paxamate (**4**) identified with shape-based screening, bifluranol (**1**), dienestrol diacetate (**5**), and p-kresalol (**2**) proposed by pharmacophore modeling, and cyqualon (**3**) suggested by docking. These results also demonstrate that, similar to the study of Krüger and Evers [30], every method on its own contributed to the identification of new COX-inhibitors.

**Fig. 7:**
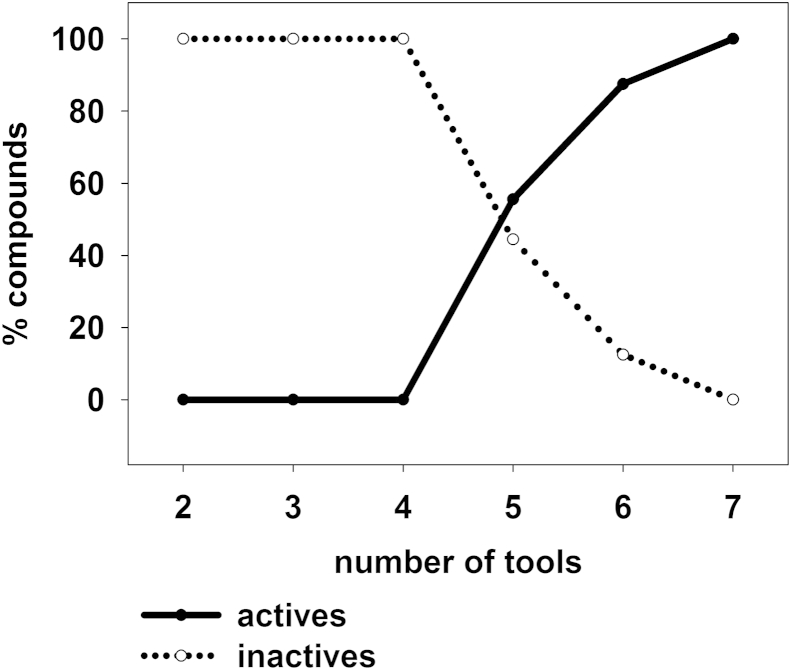
Regression analysis of consensus hits reveals a strong correlation between the biological activity and the number of predictions (R^2^ = 0.88, p = 0.0059). The percentage of active/inactive compounds in the consensus hitlist were plotted against the number of tools that predicted a compound.

Recent trends in computer-aided drug design also force the combination of methods for drug identification to increase enrichment rates [68]. In order to investigate the influence of consensus scoring on the hit rates, we calculated the percentage of biologically active and inactive compounds that have been predicted by increasing numbers of tools, and plotted it according to the biological activity (Fig. 7). For example 100% of compounds that have been predicted by all seven tools were active, while 100% of compounds that were predicted solely by two tools were inactive. Since no compound was predicted only by one method, we selected a range of two to seven tools. We performed regression analysis and observed a significant correlation between the bioactivity and the number of tools that predicted a compound (for both active and inactive compounds R^2^ = 0.88, and p = 0.0059). Therefore, the more tools predicted a compound to be active, the more likely it was active in the biological assay and *vice versa*.

In conclusion, all applied methods performed intriguingly well. We are aware of the fact that this single prospective study doesn't allow for a general conclusion concerning the applicability of the described methods, especially as we assume that different methods might be differently suitable for different targets. Also Sastry et al. have observed variances in the performance of methods depending on the target [68], and further investigations on multiple targets will be required to optimize the application of the various in silico tools. However, even in the limited scope of this study, we observed substantial differences not only in the hit rates, but also in the composition of the hitlists. Our analysis highlights the advantage of consensus methods in identifying active compounds. It is well accepted that the combination of multiple protein structures and scoring functions improves the results of docking [69,70]. Also for ligand-based methods, where consensus scoring is often referred to as data fusion, this has been proven [71,72]. However, for combinations of multiple different methods as applied here, only few studies have been published [68,73]. All studies reported in this context were conducted in a retrospective manner and to the best of our knowledge, our study is the first one showing that this also applies for prospective experiments. However, one has to be aware that a consensus approach also always represents a restriction of the original hitlist. Since every method contributed on its own to the identification of novel active molecules, the risk of missing actually active compounds rises when a consensus approach is applied. This may be of subordinate importance in a lead identification project, but during a screening for adverse events, it might be relevant.

The suitability of the tools may largely depend on various parameters like the available data, the properties of the target/ligands, the field of application, and many more, and therefore, one should carefully select the method of choice according to the project requirements.

## Figures and Tables

**Table 1:** Top-20 ranked compounds from pharmacophore modeling with descendent relative fit value.

Name	Relative fit value	Model
Picosulfate sodium	0.9677	3LN0
(R)-ibuprofen	0.9634	1Q4G
mefenamic acid	0.9629	2AYL
Dienestrol diacetate (**5**)	0.9627	3LN0
Flunixin meglumine	0.9622	2AYL
p-kresalol (**2**)	0.9585	1Q4G
Tiaprofenic acid	0.9578	1EQG
Bifluranol (**1**)	0.9568	3LN0
Meclofenamic acid	0.9561	2AYL
(S)-ibuprofen	0.9557	1Q4G
Ketoprofen	0.9556	1EQG
Oxyphenisatin acetate	0.9548	3LN0
Dormin	0.9531	2AYL
Triflocin	0.9528	1EQG
Carprofen	0.9514	1Q4G
Alclofenac	0.9475	3LN0
3, 4-dichlorobenzoic acid	0.9459	3LN0
Clofibric acid	0.9458	1Q4G
Pirprofen	0.9449	3LN0
Indometacin	0.9439	3LN0

**Table 2:** Top-20 hitlist from shape-based screening with descendent ComboScore.

Name	ComboScore	Model
Pirprofen	1.755	MF^a^
Indoprofen	1.673	MF
Tiaprofenic acid	1.658	MF
Carprofen	1.652	MF
Fenbufen	1.56	MF
Paxamate (**4**)	1.555	MF
Ketoprofen	1.532	MF
Dimetholizine	1.525	MF
(S)-ibuprofen	1.508	MF
Sulthiame	1.489	MF
17α-ethinylestradiol	1.485	MF
p-phenylacetanilide	1.481	MF
Norethynodrel	1.47	MF
Thiamine monochloride	1.468	MF
Sulfamidopyrin	1.467	MF
17α-ethinylestradiol-3-methylether	1.467	MF
(R)-ibuprofen	1.464	MF
Ticrynafen	1.447	MF
Picosulfate sodium	1.364	C^b^
Triphenyltetrazol	1.349	C

a MF methyl ester flurbiprofen model.

b C celecoxib model.

**Table 3:** Top-20 ranked compounds from docking with descendent GoldScore.

Name	GoldScore
Sulfoxone	86.49
Picosulfate sodium	80.58
Azosemide	77.55
Acid orange 6	75.35
Chloramphenicol	74.42
Fosfestrol	72.38
Bisoprolol	72.33
Ronifibrate	69.80
Baludon	69.00
Ranitidine	68.80
Glafenine	68.25
Furosemid	68.20
Thyroxin	67.83
Benzothiazide	67.67
4-deoxypyridoxine-5-phosphate	67.21
Cyqualon (**3**)	67.02
Pitofenone	66.74
Sulfasalazin	66.59
Berberine	66.31
Flavoxate	66.10

**Table 4:** All predictions of pharmacophore modeling (PM), shape-based modeling (SHAPE), Docking (DOCK), SEA, PASS, PharmMapper, and the PharmaDB for the compounds in the merged hitlist. Compounds ranked among the top-20 are highlighted in gray.

Compound name	PM^a^	SHAPE^b^	DOCK^c^	PharmaDB^a^	PharmMapper^a^	SEA^d^	PASS^e^	Activity
17α-ethinylestradiol	–f	1.49	–	0.91	–	–	–	Inactive
17α-ethinylestradiol-3-methylether	–	1.47	–	0.91	–	–	–	Inactive
3,4-dichlorobenzoic acid	0.9459	1.04	–	0.22	0.4222	–	0.535	Inactive
4-deoxypyridoxine-5-phosphate	0.8286	1.09	67.21	0.78	–	–	–	Identity not given
Acid orange 6	–	1.31	75.35	–	–	–	–	Inactive
Alclofenac	0.9475	1.32	53.92	0.81	0.5556	–	0.511	Active [40]
Azosemide	–	1.24	77.55	0.69	–	–	0.546	Inactive
Baludon	–	1.20	69.00	–	–	–	–	Inactive
Benzothiazide	–	1.15	67.67	0.60	–	–	0.574	Inactive
Berberin	–	1.33	66.31	0.55	0.5133	–	–	Inactive [50]
Bifluranol (**1**)	0.9568	1.08	–	0.43	0.5387	–	0.594	Active
Bisoprolol	–	1.00	72.33	0.63	–	–	–	Inactive
Carprofen	0.9514	1.65	62.66	0.84	0.4392	4.44E-12	0.882	Active [51]
Chloramphenicol	–	1.12	74.42	–	–	–	–	Inactive
Clofibric acid	0.9458	1.22	45.29	0.83	–	6.54E-19	0.635	Inactive
Cyqualon (**3**)	–	1.04	67.02	0.73	0.4759	5.14E-04	0.63	Active
Dienestrol diacetate (**5**)	0.9627	1.16	58.40	0.13	–	6.06E-18	–	Active
Dimetholizine	–	1.53	53.68	0.86	–	–	–	Inactive
Dormin	0.9531	1.05	41.34	0.81	–	–	–	Not available
Fenbufen	0.8274	1.56	57.55	0.01	–	–	0.57	Active [52]
Flavoxate	–	1.10	66.10	0.03	–	–	–	Identity not given
Flunixin meglumine	0.9622	1.11	47.85	0.69	0.61	–	–	Active [53]
Fosfestrol	0.9435	1.04	72.38	0.67	–	–	–	Inactive
Furosemid	–	1.28	68.20	0.44	0.50	–	–	Inactive
Glafenine	–	1.02	68.25	0.70	–	2.61E-05	–	Inactive
Indometacin	0.9439	1.35	57.47	0.72	0.65	1.80E-79	0.95	Active [54]
Indoprofen	0.9394	1.67	57.54	0.85	0.42	8.87E-13	0.65	Active [55]
Ketoprofen	0.9556	1.53	58.98	0.89	0.45	1.10E-31	0.90	Active [56]
Levothyroxin	–	1.40	67.83	0.08	–	–	–	Inactive
Meclofenamic acid	0.9561	1.08	52.10	0.76	0.48	1.89E-26	0.79	Active [57]
Mefenamic acid	0.9629	1.08	51.91	0.78	–	6.44E-25	0.60	Active [58]
p-phenylacetanilide	–	1.48	46.83	–	–	–	0.60	Inactive
Norethynodrel	–	1.47	–	0.76	–	–	–	Inactive
Oxyphenisatin acetate	0.9548	1.17	–	0.00^g^	–	3.06E-15	–	Inactive
Paxamate (**4**)	–	1.56	51.15	0.80	0.37	6.79E-18	0.53	Active
Picosulfate sodium	0.9677	1.36	80.58	0.74	–	–	–	Inactive
Pirprofen	0.9449	1.76	57.77	0.86	–	1.97E-12	0.64	Active [59]
Pitofenone	–	1.09	66.74	0.58	66.74			Inactive
p-kresalol (**2**)	0.9585	1.11	50.66	0.59	–	5.54E-18	–	Active
Ranitidine	–	1.02	68.80	0.86	0.54	–	–	Inactive
(R)-ibuprofen	0.9634	1.46	55.24	0.89	0.46	7.71E-15	0.88	Active [60]
Ronifibrate	–	1.18	69.80	0.87	0.65	–	0.53	Inactive
(S)-ibuprofen	0.9557	1.51	56.26	0.89	–	7.71E-15	0.88	Active [61]
Sulfamidopyrine	–	1.47	52.63	0.32	0.56	–	–	Not available
Sulfasalazin	–	1.23	66.59	–	–	–	0.61	Identity not given
Sulfoxone	–	1.00	86.49	–	–	–	–	Inactive
Sulthiame	–	1.49	61.94	–	–	–	0.64	Inactive
Thiamine monochloride	–	1.47	59.28	–	0.51	–	–	Inactive
Tiaprofenic acid	0.9578	1.66	58.91	0.84	–	2.17E-12	0.69	Active [55]
Ticrynafen	0.9391	1.45	56.34	0.82	–	–	0.54	Inactive
Triflocin	0.9528	1.11	49.18	0.77	–	1.51E-11	–	Identity not given
Triphenyltetrazol	-	1.35	54.36	0.90	0.53	-	0.59	Inactive

The bestfit value is depicted in case more than one was obtained.

a Relative pharmacophore fit value.

b The ComboScore was only reported above the defined activity cut-off of ≥1.00.

c The GoldScore is only reported above the defined activity cut-off of 40.00.

d The E-value is only reported below the defined activity cut-off of ≤−4.

e The Pa-value is only reported above the defined activity cut-off of ≥0.5.

f The compound was either not predicted to be active by the respective method, or above (SEA)/below (all other methods) the defined activity cut-off.

g Exact value 0.000213.

**Table 5:** IC_50_ values of the tested and active compounds.

Compound	COX-1 (μM)	COX-2 (μM)
Bifluranol (**1**)	33.0 ± 7.3	–a
Cyqualon (**3**)	1.5 ± 2.1	–
Dienestrol diacetate (**5**)	4.2 ± 1.5	2.0 ± 0.9
Paxamate (**4**)	17.1 ± 3.1	–
p-kresalol (**2**)	37.6 ± 6.8	18.2 ± 3.8
Ibuprofen (control)	3.3 ± 0.3	0.7 ± 0.4

a Inactive.

## Appendix A Supplementary data

The smiles codes of the dataset compounds used for model refinement and theoretical validation, the screening results of the inactive compounds from biological testing, and a comprehensive discussion of the novel COX-inhibitors are provided in the Supporting Information.
