# Supplementary material for: Prospective performance evaluation of selected common virtual screening tools. Case study: Cyclooxygenase (COX) 1 and 2
Source: Eur J Med Chem. 2015 May 26;96:445–57. doi: 10.1016/j.ejmech.2015.04.017 (PMC4444576; doi:10.1016/j.ejmech.2015.04.017)
Supplement: Supplementary file 1 [file mmc1.pdf]

Supporting Information

Prospective performance evaluation of selected common virtual screening tools for classical enzymes applied on the example cyclooxygenase (COX) 1 and 2

*Teresa Kaserer[a], Veronika S. Temml[a], Zsofia Kutil[b,c], Tomas Vanek[b], Premysl Landa[b], Daniela Schuster[a]**

[a]Institute of Pharmacy / Pharmaceutical Chemistry and Center for Molecular Biosciences Innsbruck, University of Innsbruck, Innrain 80-82, 6020 Innsbruck, Austria

[b]Laboratory of Plant Biotechnologies, Institute of Experimental Botany AS CR. v.v.i., Rozvojova 263, 165 02 Prague 6 - Lysolaje, Czech Republic

[c]Department of Crop Sciences and Agroforestry, Faculty of Tropical AgriSciences, Czech University of Life Sciences Prague, Kamycka 129, 165 21 Prague 6 – Suchdol, Czech Republic.

* E-mail: Daniela.Schuster@uibk.ac.at, Phone: +43 512 507 58253

Table A. Structure and biological data of all tested inactive compounds

**structure**	**name**	**% inhibition at 20µM**
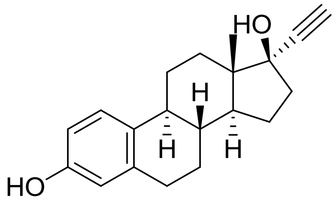	17α-ethinylestradiol	COX-1: 0.4 ± 16.7<br>COX-2: -5.2 ± 11.6

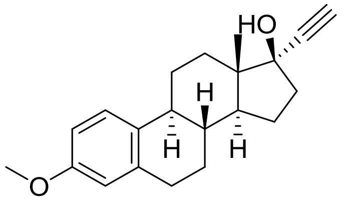	17α-ethinylestradiol-3-methylether	COX-1: 2.6 ± 11.9<br>COX-2: -10.9 ± 31.4
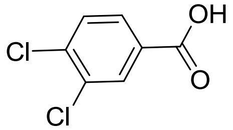	3,4-dichlorobenzoic acid	COX-1: -1.8 ± 14.3<br>COX-2: 0.1 ± 3.3
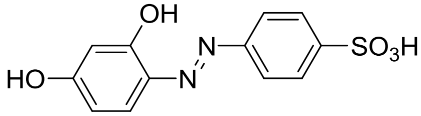	acid orange 6	COX-1: -1.2 ± 6.0<br>COX-2: -0.1 ± 5.6
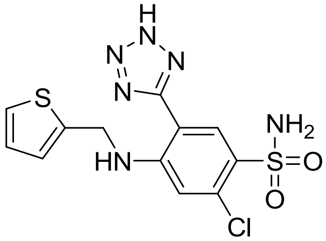	azosemide	COX-1: -9.9 ± 25.6<br>COX-2: -1.1 ± 4.7
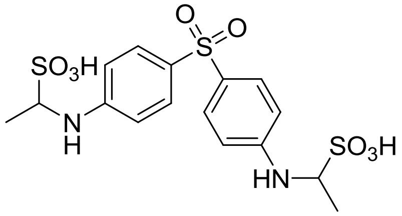	baludon	COX-1: -8.4 ± 8.7<br>COX-2: -3.7 ± 15.4
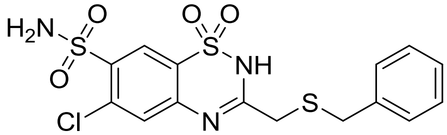	benzothiazide	COX-1: 16.6 ± 18.6<br>COX-2: -7.4 ± 25.8
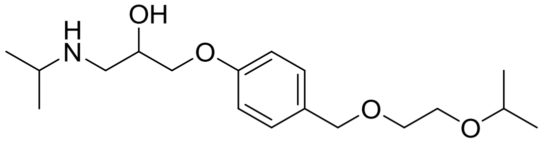	bisoprolol	COX-1: -1.4 ± 7.7<br>COX-2: 10.2 ± 7.0
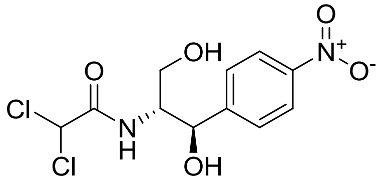	chloramphenicol	COX-1: -1.7 ± 9.5<br>COX-2: 10.7 ± 10.7
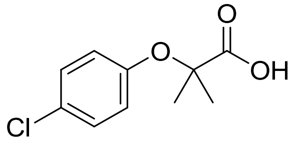	clofibric acid	COX-1: 36.4 ± 17.4<br>COX-2: 7.1 ± 18.7
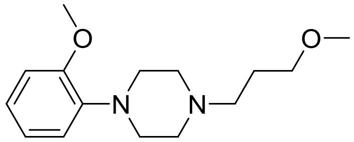	dimetholizine	COX-1: -15.1 ± 23.4<br>COX-2: -8.7 ± 8.2

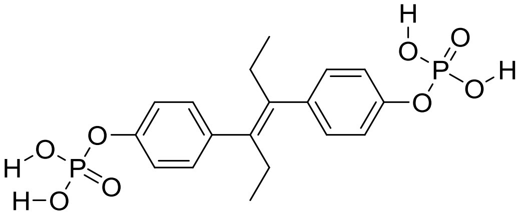	fosfestrol	COX-1: 29.1 ± 9.7<br>COX-2: 26.0 ± 5.0
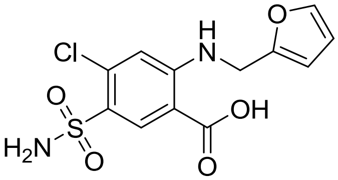	furosemid	COX-1: -1.07 ± 17.3<br>COX-2: 8.7 ± 8.9
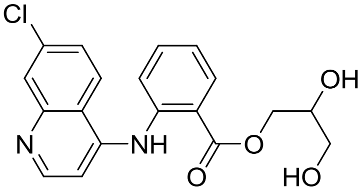	glafenine	COX-1: 3.7 ± 34.6<br>COX-2: 3.5 ± 6.0
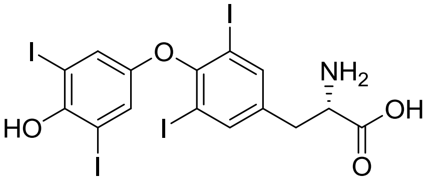	levothyroxine	COX-1: 12.9 ± 10.3<br>COX-2: 14.9 ± 18.3
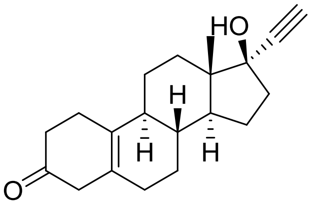	norethynodrel	COX-1: 21.0 ± 10.9<br>COX-2: -11.6 ± 32.2
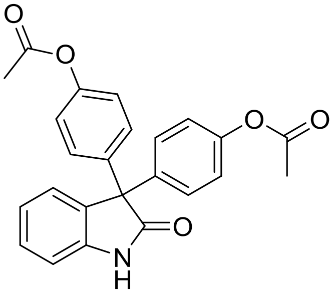	oxyphenisatin acetat	COX-1: -3.2 ± 27.9<br>COX-2: -1.8 ± 11.1
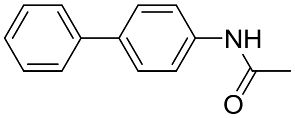	p-phenylacetanilide	COX-1: 5.8 ± 18.3<br>COX-2: -1.6 ± 8.3

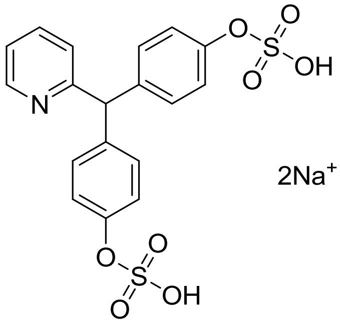	picosulfate sodium	COX-1: 8.3 ± 21.0<br>COX-2: -11.0 ± 29.4
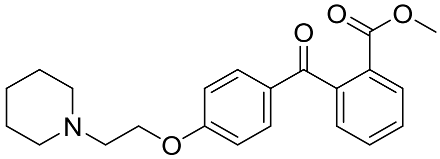	pitofenone	COX-1: -4.7 ± 16.2<br>COX-2: 14.1 ± 18.4
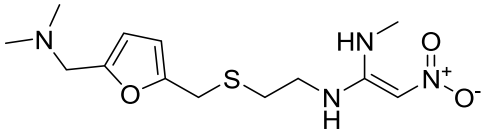	ranitidine	COX-1: -11.0 ± 9.6<br>COX-2: 12.7 ± 5.1
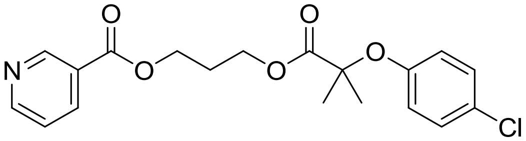	ronifibrate	COX-1: 28.2 ± 11.6<br>COX-2: 33.0 ± 5.7
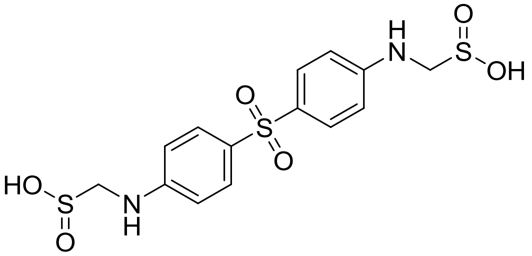	sulfoxone	COX-1: -2.1 ± 16.4<br>COX-2: -0.7 ± 6.4
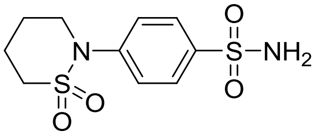	sulthiame	COX-1: 3.7 ± 9.3<br>COX-2: -0.9 ± 13.6
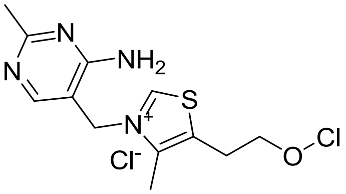	thiamine monochloride	COX-1: 13.8 ± 15.6<br>COX-2: -3.2 ± 6.9
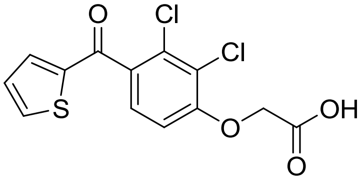	ticrynafen	COX-1: -6.2 ± 6.1<br>COX-2: 18.4 ± 6.1

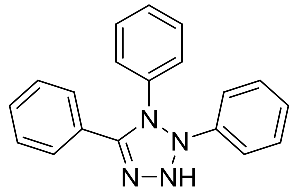	triphenyltetrazol	COX-1: 13.6 ± 14.4<br>COX-2: 2.9 ± 7.3

Comprehensive discussion of the novel COX-inhibitors

Several of the novel COX-inhibitors identified in this study belong to the class of SERMs, and have been previously described in the context of hormone-sensitive cancer. Bifluranol (**1**) is a synthetic estrogen that has anti-prostatic effects in a comparable extent to diethylstilbestrol (DES) in mice and rats. In contrast, it exhibits an approximately 8-fold lower estrogenicity compared to DES. It might have beneficial effects compared to DES, because its effects were fully reversible, and unlike DES, it did not impair spermatogenesis or fertility [1].

Research on dienestrol diacetate (**5**) was mainly performed during the seventies and focused on the beneficial outcome as feeding additives in meat and egg production. Several studies investigated the effects on gain of body weight [2] and the increase in productivity of chicken in respect to laying eggs [3], although the results appeared to be disillusioning [4].

COX-inhibition was often linked to gastrointestinal and other side effects like abdominal pain [5, 6], e.g. chlorotrianisene. However, modulation of multiple targets can also have beneficial effects, especially in complex diseases like cancer [7]. COX-2 emerges as new cancer target [8, 9] and overexpression of the inducible isoform was observed in a variety of different tumors, including stomach, esophagus, liver, pancreas, neck lung, breast, prostate, and bladder [10]. COX-1, the constitutive isoform, has long been considered to have minor influence in disease progression; however, increased levels have also been detected in ovarian [11] and cervical [12] cancer. Inhibition of COX has been proved beneficial in reducing the risk of multiple neoplastic diseases including lung, prostate, breast, and colon cancer [13], and positive effects of co-administration of

COX-inhibitors to conventional chemotherapeutics have been reported [8]. In addition, there is a cross-regulation between the estrogen and the COX pathway and ongoing efforts are made to investigate the underlying mechanisms [14] and the role of COX in hormone-sensitive cancer [15, 16].

Having in mind the beneficial effects of synthetic estrogens in hormone-sensitive cancer and the emerging role of COX in neoplastic diseases, a compound like dienestrol diacetate (**5**), that is both an estrogen receptor modulator and an unselective COX-inhibitor with an IC_{50} value comparable to ibuprofen, might have substantial advantages and should be investigated further.

Cyqualon (**3**) was identified in a screen for synthetic curcumin analogues with improved bioavailability [17]. Despite the remarkable effects of curcumin in vitro, the in vivo effects are moderate due its pharmacokinetic properties [18]. Continuous efforts are made to further investigate the biological activity of cyqualon (**3**), since multiple targets and effects were discovered. These include inhibition of p38 mitogen activated protein kinase, c-Jun N-terminal kinase, extracellular-signal regulated receptor1/2, nuclear factor ƙb, activator protein-1 transcription factor [19], p300 histone acetyltransferase [20], α-glucosidase [21], the transcriptional regulation of target gene expression in a prostate cancer cell line [22], and anti-inflammatory [23], antibacterial [24], antiproliferative and proapoptotic [25] properties. The COX-inhibition reported in this study could explain the underlying mechanism for the aniti-inflammatory effects of cyqualon (**3**).

Paxamate (**4**) and p-kresalol (**2**) are not very well investigated compounds, and since their IC_{50} values are relatively high compared to the established COX-inhibitors, the use of these compounds may be limited.

References:

[1] J.B. Dekanski, Anti-prostatic activity of bifluranol, a fluorinated bibenzyl, Br. J. Pharmacol., 71 (1980) 11-16.
[2] G.M. Herrick, J.L. Fry, B.L. Damron, R.H. Harms, Evaluation of dienestrol diacetate (Lipamone) supplementation of broiler finisher feeds on pigmentation, growth characteristics and market quality, Poult. Sci., 49 (1970) 222-225.
[3] C.R. Douglas, R.H. Harms, M.D. Carpenter, T.B. Chaille, Performance of Leghorn type hens fed two levels of energy and a synthetic estrogen during the growing period, Poult. Sci., 68 (1989) 825-829.
[4] R.H. Roberson, T. Trujillo, The effect of methionine, thiouracil, dienestrol diacetate and thyroprotein on the development and prevention of fatty liver in pullets, Poult. Sci., 54 (1975) 715-721.
[5] H. Suleyman, Y. Demircan B Fau - Karagoz, Y. Karagoz, Anti-inflammatory and side effects of cyclooxygenase inhibitors, Pharmacol. Rep., 59 (2007) 247-258.
[6] J. Bowes, A.J. Brown, J. Hamon, W. Jarolimek, A. Sridhar, G. Waldron, S. Whitebread, Reducing safety-related drug attrition: the use of in vitro pharmacological profiling, Nat. Rev. Drug Discovery, 11 (2012) 909-922.
[7] A. Petrelli, S. Giordano, From single- to multi-target drugs in cancer therapy: when aspecificity becomes an advantage, Curr. Med. Chem., 15 (2008) 422-432.
[8] J.B. Méric, S. Rottey, K. Olaussen, J.C. Soria, D. Khayat, O. Rixe, J.P. Spano, Cyclooxygenase-2 as a target for anticancer drug development, Crit. Rev. Oncol. Hematol., 59 (2006) 51-64.
[9] D.G. Menter, R.L. Schilsky, R.N. DuBois, Cyclooxygenase-2 and cancer treatment: understanding the risk should be worth the reward, Clin. Cancer Res., 16 (2010) 1384-1390.
[10] D.J. de Groot, E.G. de Vries, H.J. Groen, S. de Jong, Non-steroidal anti-inflammatory drugs to potentiate chemotherapy effects: from lab to clinic, Crit. Rev. Oncol. Hematol., 61 (2007) 52-69.
[11] R.A. Gupta, L.V. Tejada, B.J. Tong, S.K. Das, J.D. Morrow, S.K. Dey, R.N. DuBois, Cyclooxygenase-1 is overexpressed and promotes angiogenic growth factor production in ovarian cancer, Cancer Res., 63 (2003) 906-911.
[12] K.J. Sales, A.A. Katz, B. Howard, R.P. Soeters, R.P. Millar, H.N. Jabbour, Cyclooxygenase-1 is up-regulated in cervical carcinomas: autocrine/paracrine regulation of cyclooxygenase-2, prostaglandin e receptors, and angiogenic factors by cyclooxygenase-1, Cancer Res., 62 (2002) 424-432.
[13] R.E. Harris, Cyclooxygenase-2 (cox-2) blockade in the chemoprevention of cancers of the colon, breast, prostate, and lung, Inflammopharmacology, 17 (2009) 55-67.
[14] J.A. Richards, T.A. Petrel, R.W. Brueggemeier, Signaling pathways regulating aromatase and cyclooxygenases in normal and malignant breast cells, J. Steroid Biochem. Mol. Biol., 80 (2002) 203-212.
[15] E.S. Díaz-Cruz, R.W. Brueggemeier, Interrelationships between cyclooxygenases and aromatase: unraveling the relevance of cyclooxygenase inhibitors in breast cancer, Anti-Cancer Agents Med. Chem., 6 (2006) 221-232.
[16] R.W. Brueggemeier, E.S. Díaz-Cruz, P.K. Li, Y. Sugimoto, Y.C. Lin, C.L. Shapiro, Translational studies on aromatase, cyclooxygenases, and enzyme inhibitors in breast cancer, J. Steroid Biochem. Mol. Biol., 95 (2005) 129-136.

[17] B.K. Adams, E.M. Ferstl, M.C. Davis, M. Herold, S. Kurtkaya, R.F. Camalier, M.G. Hollingshead, G. Kaur, E.A. Sausville, F.R. Rickles, J.P. Snyder, D.C. Liotta, M. Shoji, Synthesis and biological evaluation of novel curcumin analogs as anti-cancer and anti-angiogenesis agents, Bioorg. Med. Chem., 12 (2004) 3871-3883.
[18] G. Shoba, D. Joy, T. Joseph, M. Majeed, R. Rajendran, P.S. Srinivas, Influence of piperine on the pharmacokinetics of curcumin in animals and human volunteers, Planta Med., 64 (1998) 353-356.
[19] C.L. Tham, K.W. Lam, R. Rajajendram, Y.K. Cheah, M.R. Sulaiman, N.H. Lajis, M.K. Kim, D.A. Israf, The effects of a synthetic curcuminoid analogue, 2,6-bis-(4-hydroxyl-3-methoxybenzylidine)cyclohexanone on proinflammatory signaling pathways and CLP-induced lethal sepsis in mice, Eur. J. Pharmacol., 652 (2011) 136-144.
[20] R. Costi, R. Di Santo, M. Artico, G. Miele, P. Valentini, E. Novellino, A. Cereseto, Cinnamoyl compounds as simple molecules that inhibit p300 histone acetyltransferase, J. Med. Chem., 50 (2007) 1973-1977.
[21] Z.Y. Du, R.R. Liu, W.Y. Shao, X.P. Mao, L. Ma, L.Q. Gu, Z.S. Huang, A.S. Chan, Alpha-glucosidase inhibition of natural curcuminoids and curcumin analogs, Eur. J. Med. Chem., 41 (2006) 213-218.
[22] K. Shoulars, M.A. Rodriguez, T. Thompson, B.M. Markaverich, Regulation of cell cycle and RNA transcription genes identified by microarray analysis of PC-3 human prostate cancer cells treated with luteolin, J. Steroid Biochem. Mol. Biol., 118 (2010) 41-50.
[23] C.L. Tham, C.Y. Liew, K.W. Lam, A.S. Mohamad, M.K. Kim, Y.K. Cheah, Z.A. Zakaria, M.R. Sulaiman, N.H. Lajis, D.A. Israf, A synthetic curcuminoid derivative inhibits nitric oxide and proinflammatory cytokine synthesis, Eur. J. Pharmacol., 628 (2010) 247-254.
[24] T. Haukvik, E. Bruzell, S. Kristensen, H.H. Tønnesen, A screening of curcumin derivatives for antibacterial phototoxic effects studies on curcumin and curcuminoids. XLIII, Pharmazie, 66 (2011) 69-74.
[25] X. Wei, Z.Y. DU, X.X. Cui, M. Verano, R.Q. Mo, Z.K. Tang, A.H. Conney, X. Zheng, K. Zhang, Effects of cyclohexanone analogues of curcumin on growth, apoptosis and NF-κB activity in PC-3 human prostate cancer cells, Oncol. Lett., 4 (2012) 279-284.
